# 1,2-Bis(bromo­meth­yl)-4,5-dimethoxy­benzene

**DOI:** 10.1107/S1600536809030001

**Published:** 2009-08-08

**Authors:** Feng-yan Zhou

**Affiliations:** a Department of Chemistry, Zaozhuang University, Shandong, People’s Republic of China

## Abstract

Colourless crystals of the title compound, C_10_H_12_Br_2_O_2_, were synthesized from 1,2-dimethoxy­benzene. The crystal structure is stabilized by inter­molecular C—H⋯O hydrogen bonds.

## Related literature

For the use of the title compound in the preparation of crown ether derivatives and isoindoline compounds, see: Dalence-Guzman *et al.* (2008 ▶); Diederich *et al.* (1993 ▶); Walpole *et al.* (1994 ▶).
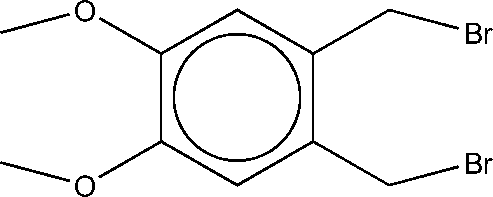


## Experimental

### 

#### Crystal data

C_10_H_12_Br_2_O_2_
*M* *_r_* = 324.00
Orthorhombic, 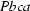

*a* = 8.125 (6) Å
*b* = 14.689 (10) Å
*c* = 20.353 (13) Å
*V* = 2429 (3) Å^3^
*Z* = 8
Mo *K*α radiation
μ = 6.65 mm^−1^
*T* = 153 K
0.15 × 0.10 × 0.10 mm

#### Data collection

Bruker APEXII CCD area-detector diffractometer
Absorption correction: multi-scan (*SADABS*; Bruker, 2005 ▶) *T*_min_ = 0.456, *T*_max_ = 0.516
12237 measured reflections
2475 independent reflections
1643 reflections with *I* > 2σ(*I*)
*R*_int_ = 0.043

#### Refinement

*R*[*F* ^2^ > 2σ(*F* ^2^)] = 0.040
*wR*(*F* ^2^) = 0.117
*S* = 1.02
2475 reflections
129 parameters
H-atom parameters constrained
Δρ_max_ = 0.89 e Å^−3^
Δρ_min_ = −0.72 e Å^−3^

### 

Data collection: *APEX2* (Bruker, 2005 ▶); cell refinement: *SAINT* (Bruker, 2005 ▶); data reduction: *SAINT*; program(s) used to solve structure: *SHELXS97* (Sheldrick, 2008 ▶); program(s) used to refine structure: *SHELXL97* (Sheldrick, 2008 ▶); molecular graphics: *ORTEP-3 for Windows* (Farrugia, 1997 ▶); software used to prepare material for publication: *WinGX* (Farrugia, 1999 ▶).

## Supplementary Material

Crystal structure: contains datablocks I, global. DOI: 10.1107/S1600536809030001/jh2086sup1.cif

Structure factors: contains datablocks I. DOI: 10.1107/S1600536809030001/jh2086Isup2.hkl

Additional supplementary materials:  crystallographic information; 3D view; checkCIF report

## Figures and Tables

**Table 1:** Hydrogen-bond geometry (Å, °)

*D*—H⋯*A*	*D*—H	H⋯*A*	*D*⋯*A*	*D*—H⋯*A*
C8—H8*A*⋯O2^i^	0.99	2.48	3.373 (6)	150
C10—H10*C*⋯O2^ii^	0.98	2.48	3.392 (7)	155

Symmetry codes: (i) 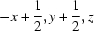; (ii) 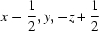.

**Fig. 1.**
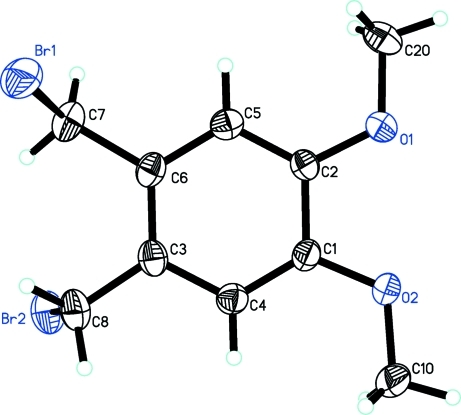
The molecular structure with atom labels and 30% probability displacement ellipsoids for non-H atoms.

**Fig. 2.**
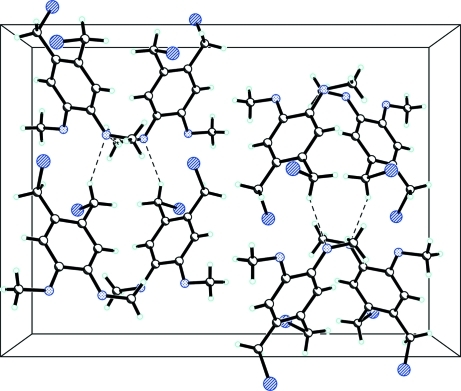
The packing diagram of molecular, viewed down the a axis, with the C—H···O interactions shown as dashed lines.

## supplementary crystallographic information

### Comment

Bis-bromomethylation of 1,2-dimethoxybenzene afforded the title compound(I), which was useful for the preparation of crown ether derivatives and isoindoline compounds (Diederich *et al.*, 1993; Walpole *et al.*, 1994; Dalence-Guzman *et al.*, 2008). It had been believed difficult to introduce hydroxyl groups directly to the 5- and 6-positions of isoindoline. With I as an intermediate, novel isoindoline derivatives could be easily prepared. The crystal structure of I is stabilized by intermolecular C–H···O hydrogen bonds.

### Experimental

Thirty-three percent HBr in AcOH (31.0 ml) was added to a solution of 1,2-dimethoxybenzene (10 g, 0.0725 mmol) and paraformaldehyde (4.35 g, 0.145 mmol) in acetic acid (100 ml), while the temperature was kept at 283 K. After stirring at room temperature for 20 h, the mixture was heated to 338 K for 1 h. The mixture was concentrated. EtOAc was added to get a white precipitate. The precipitate was filtered and washed with EtOAc to afford the title compound (9.72 g, 41.4%) as a white solid. Colourless crystals were obtained by vapor diffusion of pentane into a dichloromethane solution over a period of 3 days. ^1^H NMR (400 MHz, CDCl_3_, 295 K): 6.84 (2*H*, s), 4.63 (4*H*, s), 3.90 (6*H*, s).

### Refinement

All H atoms were placed in calculated positions and refined as riding, with C—H = 0.96–0.98 Å, and *U*_iso_(H) = 1.2–1.5 *U*_eq_(C).

### Crystal data

**Table:**

C_10_H_12_Br_2_O_2_	*F*(000) = 1264
*M**_r_* = 324.00	*D*_x_ = 1.772 Mg m^−^^3^
Orthorhombic, *P**b**c**a*	Mo *K*α radiation, λ = 0.71073 Å
Hall symbol: -P 2ac 2ab	Cell parameters from 2795 reflections
*a* = 8.125 (6) Å	θ = 2.8–22.0°
*b* = 14.689 (10) Å	µ = 6.65 mm^−^^1^
*c* = 20.353 (13) Å	*T* = 153 K
*V* = 2429 (3) Å^3^	Prism, colourless
*Z* = 8	0.15 × 0.10 × 0.10 mm

### Data collection

**Table:**

Bruker APEXII CCD area-detector diffractometer	2475 independent reflections
Radiation source: fine-focus sealed tube	1643 reflections with *I* > 2σ(*I*)
graphite	*R*_int_ = 0.043
φ and ω scans	θ_max_ = 26.4°, θ_min_ = 2.0°
Absorption correction: multi-scan (*SADABS*; Bruker, 2005)	*h* = −10→6
*T*_min_ = 0.456, *T*_max_ = 0.516	*k* = −18→17
12237 measured reflections	*l* = −25→20

### Refinement

**Table:**

Refinement on *F*^2^	Primary atom site location: structure-invariant direct methods
Least-squares matrix: full	Secondary atom site location: difference Fourier map
*R*[*F*^2^ > 2σ(*F*^2^)] = 0.040	Hydrogen site location: inferred from neighbouring sites
*wR*(*F*^2^) = 0.117	H-atom parameters constrained
*S* = 1.01	*w* = 1/[σ^2^(*F*_o_^2^) + (0.0542*P*)^2^ + 2.9265*P*] where *P* = (*F*_o_^2^ + 2*F*_c_^2^)/3
2475 reflections	(Δ/σ)_max_ < 0.001
129 parameters	Δρ_max_ = 0.89 e Å^−^^3^
0 restraints	Δρ_min_ = −0.72 e Å^−^^3^

### Special details

**Table:**

Geometry. All e.s.d.'s (except the e.s.d. in the dihedral angle between two l.s. planes) are estimated using the full covariance matrix. The cell e.s.d.'s are taken into account individually in the estimation of e.s.d.'s in distances, angles and torsion angles; correlations between e.s.d.'s in cell parameters are only used when they are defined by crystal symmetry. An approximate (isotropic) treatment of cell e.s.d.'s is used for estimating e.s.d.'s involving l.s. planes.
Refinement. Refinement of *F*^2^ against ALL reflections. The weighted *R*-factor *wR* and goodness of fit *S* are based on *F*^2^, conventional *R*-factors *R* are based on *F*, with *F* set to zero for negative *F*^2^. The threshold expression of *F*^2^ > σ(*F*^2^) is used only for calculating *R*-factors(gt) *etc*. and is not relevant to the choice of reflections for refinement. *R*-factors based on *F*^2^ are statistically about twice as large as those based on *F*, and *R*- factors based on ALL data will be even larger.

### Fractional atomic coordinates and isotropic or equivalent isotropic displacement parameters (Å^2^)

**Table:**

	*x*	*y*	*z*	*U*_iso_*/*U*_eq_	
Br1	0.41088 (8)	0.58977 (4)	0.08751 (3)	0.0691 (2)	
Br2	−0.16982 (8)	0.43548 (4)	0.13229 (3)	0.0720 (2)	
C1	0.3186 (6)	0.2549 (3)	0.1807 (2)	0.0410 (10)	
O2	0.3599 (4)	0.1778 (2)	0.21493 (15)	0.0514 (8)	
C2	0.4121 (6)	0.2681 (3)	0.12203 (19)	0.0385 (10)	
C3	0.1717 (6)	0.3963 (3)	0.1603 (2)	0.0443 (10)	
O1	0.5203 (4)	0.1999 (2)	0.10678 (14)	0.0549 (9)	
C4	0.2023 (6)	0.3175 (3)	0.1981 (2)	0.0438 (10)	
H4	0.1399	0.3077	0.2370	0.053*	
C5	0.3861 (6)	0.3470 (3)	0.0855 (2)	0.0428 (10)	
H5	0.4500	0.3574	0.0471	0.051*	
C8	0.0391 (7)	0.4600 (3)	0.1814 (2)	0.0568 (13)	
H8A	0.0745	0.5235	0.1733	0.068*	
H8B	0.0194	0.4529	0.2291	0.068*	
C7	0.2466 (7)	0.4960 (3)	0.0628 (2)	0.0554 (12)	
H7A	0.1343	0.5206	0.0689	0.067*	
H7B	0.2602	0.4801	0.0158	0.067*	
C6	0.2675 (6)	0.4116 (3)	0.1043 (2)	0.0441 (11)	
C10	0.2760 (7)	0.1627 (4)	0.2764 (3)	0.0681 (16)	
H10A	0.2901	0.2159	0.3048	0.102*	
H10B	0.3222	0.1088	0.2980	0.102*	
H10C	0.1585	0.1529	0.2680	0.102*	
C20	0.6114 (8)	0.2081 (4)	0.0474 (3)	0.0712 (16)	
H20A	0.5362	0.2043	0.0098	0.107*	
H20B	0.6923	0.1588	0.0447	0.107*	
H20C	0.6684	0.2669	0.0467	0.107*	

### Atomic displacement parameters (Å^2^)

**Table:**

	*U*^11^	*U*^22^	*U*^33^	*U*^12^	*U*^13^	*U*^23^
Br1	0.0821 (5)	0.0486 (3)	0.0765 (4)	−0.0146 (3)	0.0161 (3)	0.0010 (2)
Br2	0.0562 (4)	0.0688 (4)	0.0911 (5)	0.0108 (3)	−0.0137 (3)	−0.0024 (3)
C1	0.048 (3)	0.035 (2)	0.040 (2)	0.002 (2)	−0.0024 (19)	0.0038 (18)
O2	0.051 (2)	0.0500 (18)	0.0532 (18)	0.0134 (15)	0.0079 (15)	0.0175 (14)
C2	0.034 (2)	0.040 (2)	0.042 (2)	0.0025 (19)	−0.0016 (19)	−0.0022 (18)
C3	0.046 (3)	0.036 (2)	0.051 (3)	0.002 (2)	−0.008 (2)	−0.0076 (19)
O1	0.062 (2)	0.0549 (19)	0.0474 (17)	0.0175 (18)	0.0145 (16)	0.0053 (15)
C4	0.050 (3)	0.043 (2)	0.039 (2)	0.001 (2)	0.000 (2)	−0.0005 (18)
C5	0.042 (3)	0.048 (2)	0.038 (2)	−0.003 (2)	−0.003 (2)	0.0009 (19)
C8	0.063 (3)	0.041 (3)	0.067 (3)	0.008 (2)	−0.008 (3)	−0.010 (2)
C7	0.058 (3)	0.045 (3)	0.063 (3)	−0.003 (2)	−0.013 (3)	0.011 (2)
C6	0.052 (3)	0.034 (2)	0.046 (2)	−0.003 (2)	−0.006 (2)	0.0016 (19)
C10	0.062 (4)	0.075 (3)	0.068 (3)	0.013 (3)	0.018 (3)	0.031 (3)
C20	0.077 (4)	0.075 (4)	0.062 (3)	0.019 (3)	0.029 (3)	−0.006 (3)

### Geometric parameters (Å, °)

**Table:**

Br1—C7	1.983 (5)	C5—C6	1.405 (6)
Br2—C8	2.002 (5)	C5—H5	0.9500
C1—C4	1.365 (6)	C8—H8A	0.9900
C1—O2	1.372 (5)	C8—H8B	0.9900
C1—C2	1.428 (6)	C7—C6	1.510 (6)
O2—C10	1.442 (6)	C7—H7A	0.9900
C2—O1	1.368 (5)	C7—H7B	0.9900
C2—C5	1.394 (6)	C10—H10A	0.9800
C3—C6	1.399 (6)	C10—H10B	0.9800
C3—C4	1.413 (6)	C10—H10C	0.9800
C3—C8	1.490 (7)	C20—H20A	0.9800
O1—C20	1.422 (6)	C20—H20B	0.9800
C4—H4	0.9500	C20—H20C	0.9800
			
C4—C1—O2	126.4 (4)	H8A—C8—H8B	108.1
C4—C1—C2	119.7 (4)	C6—C7—Br1	110.6 (3)
O2—C1—C2	114.0 (4)	C6—C7—H7A	109.5
C1—O2—C10	116.9 (4)	Br1—C7—H7A	109.5
O1—C2—C5	125.8 (4)	C6—C7—H7B	109.5
O1—C2—C1	115.7 (3)	Br1—C7—H7B	109.5
C5—C2—C1	118.5 (4)	H7A—C7—H7B	108.1
C6—C3—C4	118.5 (4)	C3—C6—C5	119.7 (4)
C6—C3—C8	122.5 (4)	C3—C6—C7	121.7 (4)
C4—C3—C8	119.0 (4)	C5—C6—C7	118.7 (4)
C2—O1—C20	117.7 (4)	O2—C10—H10A	109.5
C1—C4—C3	122.1 (4)	O2—C10—H10B	109.5
C1—C4—H4	118.9	H10A—C10—H10B	109.5
C3—C4—H4	118.9	O2—C10—H10C	109.5
C2—C5—C6	121.4 (4)	H10A—C10—H10C	109.5
C2—C5—H5	119.3	H10B—C10—H10C	109.5
C6—C5—H5	119.3	O1—C20—H20A	109.5
C3—C8—Br2	110.9 (3)	O1—C20—H20B	109.5
C3—C8—H8A	109.5	H20A—C20—H20B	109.5
Br2—C8—H8A	109.5	O1—C20—H20C	109.5
C3—C8—H8B	109.5	H20A—C20—H20C	109.5
Br2—C8—H8B	109.5	H20B—C20—H20C	109.5
			
C4—C1—O2—C10	−2.0 (7)	O1—C2—C5—C6	177.7 (4)
C2—C1—O2—C10	177.1 (4)	C1—C2—C5—C6	−2.0 (6)
C4—C1—C2—O1	−177.0 (4)	C6—C3—C8—Br2	82.9 (5)
O2—C1—C2—O1	3.8 (6)	C4—C3—C8—Br2	−97.1 (4)
C4—C1—C2—C5	2.7 (6)	C4—C3—C6—C5	2.6 (6)
O2—C1—C2—C5	−176.5 (4)	C8—C3—C6—C5	−177.4 (4)
C5—C2—O1—C20	−2.2 (7)	C4—C3—C6—C7	−177.6 (4)
C1—C2—O1—C20	177.5 (4)	C8—C3—C6—C7	2.3 (7)
O2—C1—C4—C3	178.3 (4)	C2—C5—C6—C3	−0.7 (7)
C2—C1—C4—C3	−0.8 (7)	C2—C5—C6—C7	179.6 (4)
C6—C3—C4—C1	−1.9 (7)	Br1—C7—C6—C3	96.8 (5)
C8—C3—C4—C1	178.1 (4)	Br1—C7—C6—C5	−83.5 (5)

### Hydrogen-bond geometry (Å, °)

**Table:**

*D*—H···*A*	*D*—H	H···*A*	*D*···*A*	*D*—H···*A*
C8—H8A···O2^i^	0.99	2.48	3.373 (6)	150
C10—H10C···O2^ii^	0.98	2.48	3.392 (7)	155

Symmetry codes: (i) −*x*+1/2, *y*+1/2, *z*; (ii) *x*−1/2, *y*, −*z*+1/2.
